# Zootherapeutics utilized by residents of the community Poço Dantas, Crato-CE, Brazil

**DOI:** 10.1186/1746-4269-5-21

**Published:** 2009-08-05

**Authors:** Felipe S Ferreira, Samuel V Brito, Samuel C Ribeiro, Waltécio O Almeida, Rômulo RN Alves

**Affiliations:** 1 Universidade Regional do Cariri, Departamento de Química Biológica, Crato, CE, Brazil; 2 Universidade Federal do Pernambuco, Departamento de Zoologia, Recife, PE, Brazil; 3 Universidade Estadual da Paraíba, Departamento de Biologia, 58109-753, Campina Grande, Paraíba, Brazil

## Abstract

**Background:**

Animals have been used as a source of medicine in Brazil since ancient times, and have played a significant role in healing practices. Specifically in Northeast Brazil, zootherapy is a very common practice, and together with medicinal plants, it plays an important role as a therapeutic alternative. In the state of Ceara, no works have been carried out on rural communities with regard to use of zootherapeutics, even though the practice of zootherapy is common in this region. Therefore, the aim of this study was to analyze the use of medicinal animals in a rural community (Poco Dantas) in the municipality of Crato, Ceara, Brazil.

**Methods:**

The field survey was carried out from October 2008 to January 2009 by conducting interviews using structured questionnaires with 72 people (33 men and 39 women), who provided information on animal species used as remedies, body parts used to prepare the remedies, and ailments for which the remedies were prescribed. We calculated the informant consensus factor (ICF) to determine the consensus over which species are effective for particular ailments, as well as the species use value (UV) to determine the extent of utilization of each species.

**Results:**

A total of 29 species, distributed in 17 families were categorized as having some medicinal property. The taxa most represented were: mammals (9), insects (7), reptiles and birds (4). *Progne chalybea*, a species not previously recorded as being of medicinal use, was cited in the present work, where it is utilized in the treatment of alcoholism. The animals are used in the treatment of 34 diseases or symptoms, where sore throat, inflammations and cough are the ailments with the greatest number of citations.

**Conclusion:**

The data show that zootherapy represents an important therapeutic alternative for the inhabitants of the community. New studies on medicinal fauna should be conducted with the aim of determining the exploitation level of the species utilized, promoting sustainable development of medicinal species that are eventually threatened, and preserving and disseminating the knowledge developed by traditional individuals of the community.

## Background

Wild animals and their products constitute essential ingredients in the preparation of traditional medicine [1-5]. As mentioned by Marques [6], "all human civilisations with a structured medicinal system would utilise animals as medicines." Although animal-derived remedies constitute an integral part of folk medicine in many parts of the world, particularly for people with limited or no access to mainstream medical services, their role in health care has generally been overlooked in discussions about public health, conservation and management of faunistic resources, and ecosystem protection [5].

Animals have been used as a source of medicine in Brazil since ancient times, and have played a significant role in healing practices. Many people still use animals as medicines as an alternative or supplement to visiting a modern health care practitioner [7]. Various animal species have been used for medicinal purposes in Brazil by indigenous societies, and by descendents of the European colonizers in the last four centuries [8]. Meanwhile, there are still few ethnoecological studies conducted on the topic in our country when compared to the number of existing medicinal plants. This same situation also exists in clinical pharmacological studies on the efficacy of natural products. According to Harvey [9], 225 drugs are in some stage of development from natural products, where 108 are from plant products and 24 from animals, demonstrating the need of studies that evaluate the efficacy of zootherapeutics.

Worldwide, studies on zootherapy have increased in the last years, demonstrating that this practice is common in various parts of the world, especially in Asia and Africa [4,10-19]. In Brazil, publications containing quantitative data on zootherapy have recorded that about 290 species of animals are used in Brazilian traditional medicine [5,7,8,10-12]. Specifically in Northeast Brazil, zootherapy is a very common practice [1,20,21], and together with medicinal plants, it plays an important role as a therapeutic alternative. Due to difficult access to health services, and their limitations, traditional remedies have become an inexpensive alternative which is easy to obtain [5].

In the state of Ceara, no works have been carried out on rural communities with regard to use of zootherapeutics, even though the practice of zootherapy is common in this region. Therefore, the aim of this study was to analyze the use of medicinal animals in a rural community in the municipality of Crato and to determine the following points: i) which species of medicinal animals are used by the community? ii) which parts of the animals are used? iii) which ailments are treated with animal-based remedies?

## Methods

### Study Area

The study was conducted in the community Poço Dantas, located in the Distrito de Monte Alverne (7° 07' 21" S × 39° 31' 19" W), which is 23 km from the center of the municipality of Crato, state of Ceara, close to the slope of Chapada do Araripe. The area is surrounded by vegetation of the "Mata Seca (as dry vegetation). In an area close to the slope of the plateau, there is a strip of "Mata de cocaais" (is a forest of transition, located in the northeastern Brazil, between caatinga and cerrado showing typical vegetation as: babassu and palm tree) characteristic of all the slope of Chapada do Araripe [22]. The principal economic activity of the inhabitants is agriculture. The community has only one clinic that provides care for the residents of the community Poço Dantas and of the neighboring communities.

**Figure 1:**
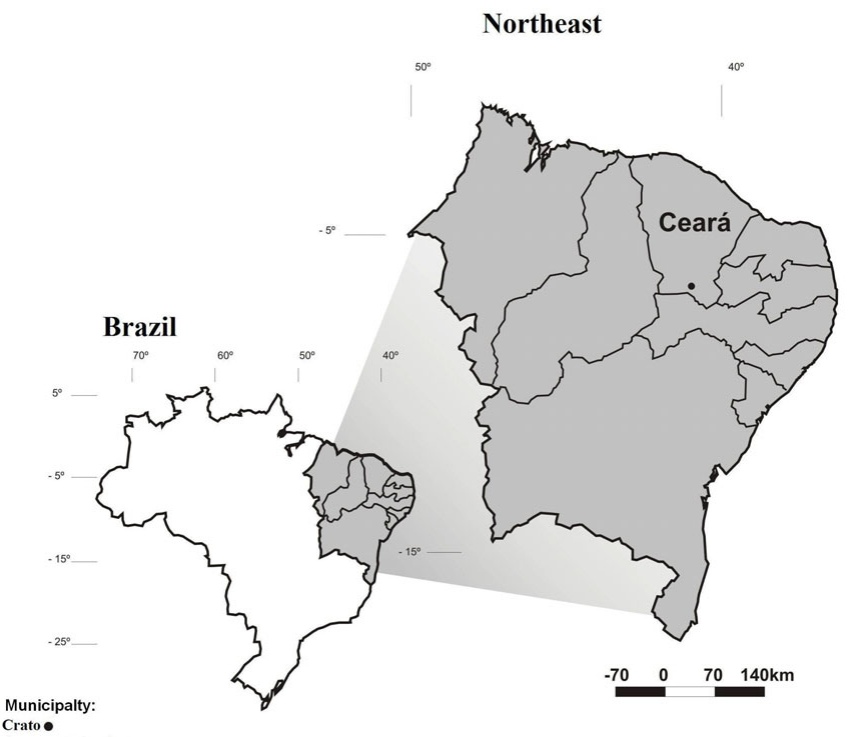
**Map locating the city studied in Ceará State, Brazil**.

The municipality of Crato occupies an area of approximately 1,009 km^2 ^and has approximately 111,000 inhabitants [22] (Figure 1). The principal economic activities of Crato are related to commerce and tourism. The municipality of Crato has six hospitals and 20 health centers [23]. Crato is located in the Araripe bio-region, which is composed of a mosaic of ecosystems including humid forests (sub-perennial tropical cloud forest), dry forests (sub-deciduous tropical rain forest), "cerradão"(sub-deciduous tropical xeromorphic forest), "caatinga" (deciduous thorn forest), and "carrasco" (xerophilous vegetation) [23,24].

The faunal composition of the Araripe bio-region includes common vertebrate taxa such as mammals [25], birds [26], and reptiles [27], while the most investigated invertebrate taxa include nematodes [28], pentastomids [29] and triatomines [30].

### Procedures

The survey was conducted in the period of October 2008 to January 2009 among the inhabitants of the community Poço Dantas. Information was obtained from 72 residents (33 men and 39 women). The age range of the interviewees varied from 22 to 78 years. Of the dwellers interviewed, 8 (11.2%) had a complete secondary education, 23 (31.9%) had a complete elementary education and 41 (56.9%) had an incomplete primary education. The residents of the community Poço Dantas had a monthly income of R$ 220,00 (US$ 422,40) to R$ 500,00 (US$ 960,00).

To respect intellectual property rights, we adopted the following protocol in the field: before the survey, we introduced ourselves, explained the nature and objectives of our research and asked the respondents for permission to record the information. The ethical approval for the study was obtained from the Ethics committee of Faculdade de Medicina de Juazeiro do Norte – FMJ (N° of protocol: 2009-0319CEP).

Sampling was non-random intentional, in which the interviewees were pre-defined [31], which consisted of all the dwellers of the community who were over 20 years of age. Semi-structured questionnaires were used, complemented by free interviews and informal conversations [32]. The questionnaires contained questions on the animal species used for medicinal purposes, their respective uses, preparations and parts utilized.

Vernacular names of species were recorded as quoted during the interviews. Zoological material was identified with the aid of specialists, through examination of voucher specimens donated by the interviewees or purchased at the surveyed markets, and through photographs taken during interviews of the animal species or their parts. Whenever necessary, these procedures were supplemented by checking vernacular names provided by traders against the scientific names, with the aid of taxonomists familiar with the study areas. All specimens that were collected, the questionnaires and as photographs of the specimens described, were deposited in the Laboratório de Zoologia da Universidade Regional do Cariri – URCA.

### Data Analysis

The ailments treated by zootherapeutics were grouped into 10 categories (Table 1) based on the model used by the "Centro Brasileiro de Classificação de Doenças" (Brazilian Center for the Classification of Diseases) [33].

**Table 1:** Categories of diseases treated with animal- based medicines by residents of the community, according to the Centro Brasileiro de Classificação de Doenças (1993)

Categories	Ailments cited by the residents	Total
A	Inflammations, inflammations of umbilical cord of newborn baby, changes of skin and alleviate tremor	4
B	Asthma, sore throat, coughs, bronchitis, expectorant, flu, pneumonia and nasal congestion	8
C	Rheumatism, arthritis, back ache, arthrosis, osteoporosis, pain in bones, healing and toothache	7
D	Cholesterol, diabetes and varices	3
E	Burns, snake bites, alcoholism and bruises	4
F	Deafness and ear aches	2
G	Urinary infections	1
H	Stomach ache	1
I	Fissures on feet	1
J	Headache	1
K	Injuries of animals (estrepada)	1

Total	34	

A: Undefined illnesses; B: Respiratory system; C: Osteomuscular system and conjunctive tissue; D: Circulatory system; E: Lesions caused by poisoning and other external causes; F: Hearing ailments; G: Diseases of the urogenital system; H: Digestive system; I: Skin and the subcutaneous tissue; J: Nervous system; K: Zootherapeutic products for veterinary medicine

To estimate the level of agreement between interviewees over which animals to use for each category, we calculated the informant consensus factor (ICF), adapted from Heinrich et al. [34] that looks at the variability of animals used for each treatment, and therefore the consensus between practitioners. This factor estimates the relationship between the "number of use-reports in each category (*n*ur) minus the number of taxa used (*n*t)" and the "number of use-reports in each category minus 1." ICF is thus calculated using the following formula:



The product of this factor ranges from 0 to 1. A high value (close to 1) indicates a high consensus, where relatively few taxa (usually species) are used by a large proportion of people, while a low value indicates that the informants disagree on the taxa to be used for treating a particular illness.

### Use-value

For each species the use-value (UV), adapted from the proposal of Phillips et al., [35], was calculated. This quantitative method evaluates the relative importance of each medicinal species based on its relative use among informants. These values were calculated using the following formula: UV = Σ*U*/*n*, where U is the number of times a species is cited and *n *is the number of informants. The use-value of each species is therefore based objectively on the importance attributed by the informants and does not depend on the opinion of the researcher.

## Results and discussion

In the area studied, the use of animals for therapeutic purposes was shown to be an important alternative to medications sold in pharmacies. A total of 29 species distributed in 17 families were reported as having some medicinal property (Table 2). The taxa most represented were: mammals (9), insects (7), reptiles and birds (4). These results reinforce the importance of studies whose aim is the identification of these folklore remedies, in order to help strategies for maintaining this traditional medicine knowledge, as well as the rational and sustainable use of biodiversity [36].

**Table 2:** Species of animals used for medical purposes in the community Poço Dantas, municipality of Crato, Ceará, Brazil

Family/species/local name	Number of citations	Use-value	Part used	Disease (or illness)
**Insects**				
Gryllidae – "Cricket" or "grilo"	2	0.02	Leg (1)	Urinary infections
Apidae *Melipona scutellaris *(Latreille, 1811) – "Stingless bee" or "uruçú"	6	0.08	Wax and honey (2)	Healing, sore throat, coughs and asthma
*Melipona subnitida *(Ducke, 1910) – "Stingless bee" or "jandaíra"	4	0.05	Honey (2)	Asthma, sore throat and coughs
*Partamona cupira *(Smith, 1863) – "Stingless bee" or "cupira"	23	0.31	Wax (2) and honey (3)	Stomach ache, ear aches, healing, sore throat, coughs, asthma and expectorant
*Apis mellifera *(Linnaeus, 1758) – "Honey bee" or "abelha italiana"	27	0.37	Honey (3)	Coughs, flu, asthma, sore throat and healing
*Trigona spinipes *(Fabricius, 1793) – "Stingless bee" or "arapuá"	4	0.05	Honey (3)	Coughs and ear aches
Termitidae "Termite" or "cupim"	2	0.02	Whole animal (4)	Asthma
**Fishes**				
Erythrinidae				
*Hoplias malabaricus *(Bloch, 1794) – "Fish" or "traíra"	15	0.2	Fat and secretion epidermal (2)	Ear aches, inflammations, cholesterol, sore throat, healing, inflammations of umbilical cord of newborn baby, bruises and alcoholism
Prochilodontidae				
*Prochilodus nigricans *Agassiz, 1929 – "Black prochilodus" or "curimatã"	9	0.12	Fat (2)	Inflammations and cholesterol
**Amphibians**				
Bufonidae				
*Rhinella jimi *(Stevaux, 2002) – "Cururu toad" or "sapo cururu"	5	0.06	Fat (2)	Bruises, inflammations, arthritis, treatment of injured animals
Leptodactylidae				
*Leptodactylus labyrinthicus *(Spix 1824) – "South american pepper frog" or "gia"	2	0.02	Fat (2)	Sore throat
**Reptiles**				
Chelidae				
*Phrynops tuberosus *(Peters, 1870) – "Side-necked turtle" or "cágado"	31	0.43	Fat (3), shell (4) and blood (2)	Sore throat, rheumatism, inflammations, fissures on feet, back ache, changes of skin, asthma, coughs, (3, 4) varices (2)
Viperidae				
*Crotalus durissus *(Linnaeus, 1758) – "Rattle snake" or "cascavel"	4	0.05	Fat (2)	Snake bites, bruises, rheumatism, inflammations, arthritis, alleviate tremor, treatment of injured animals
Iguanidae				
*Iguana iguana *(Linnaeus, 1758) – "Common iguana" or "camaleão"	10	0.13	Fat (2)	Ear aches, sore throat and inflammations
Teiidae				
*Tupinambis merianae *(Duméril and Bibron, 1839) – "Teju lizard" or "tiú"	66	0.91	Fat (2) and tail (5)	Sore throat, coughs, asthma, headache, bruises, inflammations, rheumatism, flu, bronchitis, arthritis, arthrosis, back ache, toothache, healing, fissures on feet (2) deafness and ear aches (2, 5)
**Birds**				
Cathartidae				
*Coragyps atratus *(Bechstein, 1793)- "Black vulture" or "urubu"	6	0.08	Meat and whole animal (6)	Asthma, coughs and alcoholism
Phasianidae				
*Gallus domesticus *Linnaeus, 1758 -"Chicken" or "galinha"	49	0.68	Fat (3), esophagus and stomach (7)	Sore throat, coughs, inflammations, pneumonia, ear aches, toothache, nasal congestion, stomach ache (3) e diabetes (7)
*Pavo cristatus *Linnaeus, 1758 – "Pea-cock" or "pavão"	8	0.11	Feather (4)	Asthma
Hirundinidae				
*Progne chalybea *(Gmelin, 1789) – "Gray-breasted martin" or "andorinha"	1	0.01	Whole animal (6)	Alcoholism
Cuculidae				
*Crotophaga ani *Linnaeus, 1758 – "Smooth-billed ani" or "anu"	2	0.02	Feather (4)	Asthma
*Mammals*				
Canidae				
*Cerdocyon thous *(Linnaeus, 1766) -"Fox" or "raposa"	3	0.04	Fat (3) and liver (7)	Rheumatism and bronchitis
Felidae				
*Felis silvestris *Schereber, 1775 – "Cat" or "gato"	2	0.02	Fur (2)	Healing
Dasyponidae				
*Euphractus sexcinctus *(Linnaeus, 1758) – "Armadillo" or "tatu-peba"	6	0.08	Fat (2), meat (6) and tail (5)	Rheumatism, burns, inflammations, pain in bones (2, 6), ear aches and deafness (5)
*Dasypus novemcinctus*, Linnaeus,1758 – "Nine-banded armadillo" or "tatu-galinha"	2	0.02	Fat (2), meat (6) and tail (5)	Rheumatism, burns, inflammations, pain in bones (2, 6), ear aches and deafness (5)

Bovidae				
*Ovis aries *(Linnaeus, 1758)- "Sheep" or "carneiro"	19	0.26	Fat (2)	Fissures on feet, rheumatism, arthritis, back ache, ear aches and inflammations
*Bos taurus *(Linnaeus, 1758) – "Domestic cattle" or "boi"	5	0.06	Milk (8)	Inflammations, sore throat and arthritis
*Capra hircus *(Linnaeus, 1758) – "Domestic goat" or "bode"	2	0.02	Milk (8)	Coughs and bronchitis
Suidae				
*Sus scrofa *(Linnaeus, 1758) – "Pig" or "porco"	1	0.01	Testicle (4)	Bronchitis
Equidae				
*Equus asinus *Linnaeus, 1758 – "Asino" or "jumento"	7	0.09	Milk (8)	Coughs

Legend: (1) Prepare a tea with the animal part utilized and ingest it; (2) Ingest directly or rub on the affected area; (3) Similar to item two, can be administered in combination with medicinal plants; (4) Macerate, prepare a tea and ingest; (5) Rub on the ear; (6) Cook without salt and ingest; (7) To dry under the sun and ingest with tea or with some food; (8) Ingest in combination with medicinal plants or with honey of *A. mellifera *and *P. cupira*.

**Figure 2:**
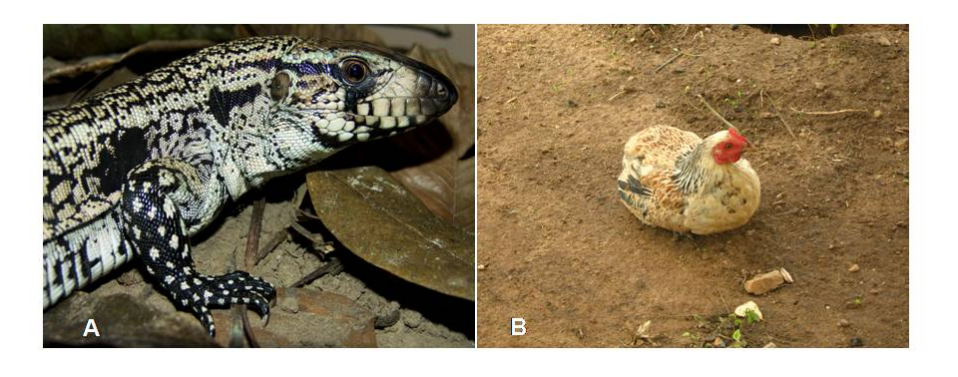
**Examples of animal used for medical purposes in the community Poço Dantas, municipality of Crato, Ceará, Brazil**. A: *Tupinambis merianae *(Photo: Daniel Loebmann), B: *Gallus domesticus *(Photo: Samuel C Ribeiro).

The species most often cited were: *Tupinambis merianae *(n = 66), *Gallus domesticus *(n = 49), *Phrynops tuberosus *(n = 31) and *Apis mellifera *(n = 27) (Figure 2). With exception of *Progne chalybea *(andorinha), all other species were recorded as being used in popular medicine in other communities of Brazil [21]. This is the first record of medicinal use for *P. chalybea*, a bird that according to the interviewees is cooked without salt and ingested, where it is used in the treatment of alcoholism.

As in the present study, ethnozoological surveys conducted in other regions of Brazil have demonstrated the importance of zootherapy in the country. In Bahia, there was a report of 23 animal species in the municipality of Remanso [37], while in Tanquinho 34 species [38]. Branch & Silva [39] reported 33 species in Alter do Chão, Para, and Begossi [40] reported the use of 10 species on Ilha de Buzios in São Paulo. Marques [41] noted the use of 56 species in Varzea de Marituba in the state of Alagoas, while Seixas & Begossi [42] reported 16 species used on Ilha Grande in Rio de Janeiro. Rodrigues [43] reported the use of 29 species in Parque Nacional de Jau, Amazonas. Alves & Rosa [7,11,12] described the use of 74 species in Mamanguape in Paraiba, 57 species in Raposa in Maranhão, 54 species on Ilha do Marajo, and 46 species in Cajueiro da Praia in Piaui.

Some of the animals recorded in the present study are also utilized in popular medicine in other countries. For example, in Mexico, *Crotalus durissus *is utilized to help in childbirth, and *Coragyps atratus *is utilized for difficulties during childbirth, lack of air, tumescence and epileptic attack [44,45]. In India, *Pavo cristatus *is used for ear infections, muscle pain and earache, *Sus scrofa *for muscle pains, rheumatism, snake bite, burns and sexual impotence, *Felis domesticus *for arthritis, and *G. domesticus *for burns [14-17]. In Sudan, *A. mellifera *is used for gastric ulcers and wounds, *Capra hircus *for burns, and diseases of mouth and of the skin, and *G. domesticus *as an aphrodisiac and for cough, while *Ovies aries *is used in the treatment of gingivitis [46]. Our results and those of the studies cited above reveal the wide dissemination of some species in different traditional medical systems throughout the world.

Some of the reported in this work have been studies in order to describe the clinical and pharmacological properties of substances isolated from animals. Temponi et al. [47] describe steroids from the skin of *Rhinella jimi *with an important antileishmanial and atitrypanosomal activity. van Dijk et al. [48] isolated a protein from de blood of *G. domesticus*, called cathelicidin, wich inhibits the growth of bacteria of the genus *Salmonella*. Park et al. [49] isolated a protein lactophoricin of the milk from *Bos taurus *that can be used against infections caused by *Escherichia coli*. Of the species recorded, the majority are represented by wild species. Only eight domestic animals were reported as being utilized for medicinal purposes, including: *G. domesticus*, *P. cristatus*, *O. aries*, *B. taurus, S. scrofa*, *C. hircus*, *Equus asinus *and *F. domesticus*. Almeida [8] states that the majority of the domestic species utilized for medicinal purposes are of European origin and not native to Brazil, which corroborates the observations of our study and provides evidence that the knowledge of traditional medicine of indigenous and/or traditional communities in the country is a result of the miscegenation of Native Americans, Africans and Europeans, as pointed out by Rodrigues [43] and Alves et al [5].

Among the species mentioned, 11 have been utilized since the 17th century by indigenous populations in Brazil. These findings show the capacity of reproduction of zootherapeutic practices over the years [8].

There were no reports of medicinal species originating from other biomes in the community studied, showing the importance of local biodiversity for the choice of medicinal animals. The local fauna, as a consequence of its availability and ease of access, influences directly the choice and use of animals for therapeutic purposes [7,50].

Various parts of the animals are utilized in the preparation of medications, including: honey, wax, milk, skin secretions, blood, organs (liver, esophagus and stomach), fat, feathers, legs, paws, tail, fur, hides, testicles and meat. Animals such as *P*. *tuberosus*, *C. atratus *and *P. chalybea *can be utilized whole. The most cited zootherapeutic product cited the most was lard. The wide utilization of lard can be attributed to the fact that the main medicinal animals utilized are vertebrates which have a large quantity of body fat [51].

According to the informants, two species are utilized in veterinary medicine. The lard of *R. jimi *and *C. durissus *is utilized in the treatment of wounds (commonly called estrepada) in the hide of horses, cattle and donkeys. Barboza et al., [52] reported the use of 15 species of medicinal animals in veterinary ethnomedicine, in the municipality of Cubati, Paraiba, Brazil. These authors also observed that lard is one of the zootherapeutic products most used in veterinary medicine.

According to the interviewees, the animal-based remedies are applied for 34 ailments (Table 1). A total of 323 citations of use were recorded (Table 3). The categories with the highest number of citations were: diseases of the respiratory system, undefined illnesses and diseases of the osteomuscular system and conjunctive tissue. Some categories showed few citations, such as: diseases of the urogenital system, diseases of the nervous system and zootherapeutic products for veterinary medicine. The low number of citations for these categories can be attributed to the fact that few species were reported for the treatment of these diseases (4 species) and species just mentioned are reported for these purposes (see Alves [21]).

**Table 3:** Consensus factors of the informants for the categories described

	Categories										
	
	A	B	C	D	E	F	G	H	I	J	K
Species used	12	19	14	4	9	8	1	3	3	1	2
Percentage of species used (%)	41.3	65.5	48.2	13.7	31.0	27.5	3.44	10.3	10.3	3.4	6.8
Use citations	69	226	69	13	21	45	2	15	16	2	4
Percentage of use citations (%)	21.3	69.9	21.3	4.02	6.5	13.9	0.61	4.6	4.9	0.6	1.2
ICF	0.83	0.92	0.8	0.75	0.6	0.84	1	0.85	0.86	1	0.66

The ailments with the most citations were: sore throat (107 citations; 33.1%), inflammations (65 citations; 20.1%) and cough (54 citations; 16.7%). Other studies conducted in Northeast Brazil also indicate that these ailments are widely treated with medicinal animals [7,11,12,37,38,53]. Some categories showed a high ICF value (see table 3). The high consensus values corroborate other works conducted of North and Northeast Brazil [7,11,12,37], which shows that the majority of animal-based remedies are utilized in the treatment of diseases of the respiratory system. Alves and Rosa [54] listed 132 species of animals utilized for the the treatment of diseases of the respiratory system.

In the categories diseases of the respiratory system, skin and the subcutaneous tissue, hearing disorders, undefined illness and diseases of the osteomuscular system and conjunctive tissue, the species with the highest use value was *T. merianae *(UV = 0.91), while in the category digestive system, the species with the highest use value was *G. domesticus *(UV = 0.68).

With respect to the preparation of the medications, the following ways could be observed: 1) whole animals or body parts are macerated, where the resultant powder is ingested in the form of teas or together with foods and 2) secretions and metabolic products (blood, skin secretions, milk and honey) and lard are administered as pumices or ingested pure or combined with medicinal plants or other animal-based products. According to the informants, milk of *E. asinus*, *B. taurus *and *C. hircus *is utilized in combination with plants or with honey from *A. mellifera *and *P. cupira*. The lard of *G. domesticus and P. tuberosus*, as well as honey from *P. cupira*, *A. mellifera *and *T. spinipes *are utilized in combination with plants. The combination of zootherapeutic and phytotherapeutic products has already been reported in other studies carried out in Northeast Brazil [7,11,12,53].

The use of some animals cited in the present study is associated with popular beliefs, also known as superstitions. For example, the rump of *E. sexcinctus*, *D. novemcinctus *and *T. merianae *is introduced in the ear to treat and/or cure deafness, a form of use also recorded in the city of Santa Cruz do Capibaribe, in the state of Pernambuco [51] and in the city of Crato and Juazeiro do Norte, in the state of Ceará [55]. These findings are in agreement with Alves et. al [5] and Alves [21] who point out that the use of animal-based products is directly associated with local beliefs in different regions of Brazil.

Among the medicinal species listed in the present study, 10 (34.4%) are also utilized as food. They are: *H. malabaricus*, *P. nigricans*, *T. merianae*, *I. iguana*, *G. domesticus*, *E. sexcinctus*, *D. novemcinctus*, *O*. aries, *B. taurus*, *C. hircus*. Alves [23] lists the medicinal and nutritional use of 175 species of animals in Northeast Brazil. These findings show the importance of biodiversity as a resource of medicinal and nutritional products, reinforcing the need for policies for the rational and sustainable use of biodiversity.

It is well known that the use of some animal species for medicinal purposes is a cause for concern for particular species [53]. In the present study, all medicinal species listed are not threatened of extinction. However, other articles published shown the medicinal use and commerce of threatened species on the Brazilian Northeastern region [56-59]. These demonstrate the need to assess the implications of the animal use in popular medicine, and the need for including such uses in discussions of animal conservation [59-61].

Considerable attention has been given to the use of natural resources of Brazil and of the world, such that ethnobiological studies are essential in helping their use and management [49]. Traditional communities have a wide natural pharmacopeia containing various animal and plant species, representing a path toward discovering new drugs through the pharmaceutical industry [3]. Therefore, ethnoscientific studies that focus on social, cultural, economic, clinical and environmental aspects of medicinal animals are important, as they are aimed at establishing measures of suitable management that make the sustainability of zootherapeutic resources possible.

In the context of sustainability of complex socio-ecological systems, the preservation of knowledge, culture and biological diversity are equally significant, and perhaps more importantly are interlinked. It is important to understand the value of wildlife to human society, to further its protection for future generations, ensuring that knowledge and culture are maintained. The medicinal value of species represents an important link between human society and biodiversity that therefore needs to be recognized in efforts aimed at sustainable use of ecosystems [62].

## Conclusion

The present work recorded the use of 29 species of animals utilized in popular medicine in the area studied. The zootherapeutic products recorded are utilized in the treatment of 34 ailments, where sore throat, inflammations and cough are the most often treated with these remedies. The data show that zootherapy represents an important therapeutic alternative for the inhabitants of the region. Further studies of medicinal fauna are warranted to: determine the level and extent that these species are utilized; guide user populations with regard to the correct use of popular medications; promote the sustainable development of potentially threatened medicinal species; preserve and disseminate knowledge acquired by individuals of the traditional community, guaranteeing the preservation of this knowledge; and identify and develop new medicines from these natural products.

## Competing interests

The authors declare that they have no competing interests.

## Authors' contributions

FSF, SVB, RRNA and WOA – Writing of the manuscript, literature survey and interpretation; FSF, SVB and SCR- Ethnozoological data, literature survey and interpretation; SCR and FSF – Analysis of taxonomic aspects. All authorsread and approved the final manuscript.
